# Selective Disruption of the Blood–Brain Barrier by Zika Virus

**DOI:** 10.3389/fmicb.2019.02158

**Published:** 2019-09-18

**Authors:** Ana Rachel Leda, Luc Bertrand, Ibolya Edit Andras, Nazira El-Hage, Madhavan Nair, Michal Toborek

**Affiliations:** ^1^Department of Biochemistry and Molecular Biology, Miller School of Medicine, University of Miami, Miami, FL, United States; ^2^Department of Immunology and Nano-Medicine, Herbert Wertheim College of Medicine, Florida International University, Miami, FL, United States

**Keywords:** Zika virus, blood–brain barrier, endothelial cells, tight junctions, neuroinfection

## Abstract

The blood–brain barrier (BBB) selectively regulates the cellular exchange of macromolecules between the circulation and the central nervous system (CNS). Here, we hypothesize that Zika virus (ZIKV) infects the brain via a disrupted BBB and altered expression of tight junction (TJ) proteins, which are structural components of the BBB. To assess this hypothesis, *in vitro* and *in vivo* studies were performed using three different strains of ZIKV: Honduras (ZIKV-H), Puerto Rico (ZIKV-PR), and Uganda (ZIKV-U). Primary human brain microvascular endothelial cells (BMECs) were productively infected by all studied ZIKV strains at MOI 0.01, and were analyzed by plaque assay, immunofluorescence for NS1 protein, and qRT-PCR at 2 and 6 days post-infection (dpi). Compared to mock-infected controls, expression level of ZO-1 was significantly upregulated in ZIKV-H-infected BMECs, while occludin and claudin-5 levels were significantly downregulated in BMECs infected by all three studied viral strains. Interestingly, BMEC permeability was not disturbed by ZIKV infection, even in the presence of a very high viral load (MOI 10). All studied ZIKV strains productively infected wild-type C57BL/J mice after intravenous infection with 10^7^ PFU. Viral load was detected in the plasma, spleen, and brain from 1 to 8 dpi. Peak brain infection was observed at 2 dpi; therefore, TJ protein expression was assessed at this time point. Claudin-5 was significantly downregulated in ZIKV-U-infected animals and the BBB integrity was significantly disturbed in ZIKV-H-infected animals. Our results suggest that ZIKV penetrates the brain parenchyma early after infection with concurrent alterations of TJ protein expression and disruption of the BBB permeability in a strain-dependent manner.

## Introduction

Neurotropic viral infection can directly and indirectly (e.g., by inducing local immune responses) disrupt the complex structural and functional architecture of the central nervous system (CNS). In addition, several neurological disorders are often associated with autoimmune mechanisms initiated by a viral infection, such as Guillain-Barré syndrome (van den Berg et al., 2014). Neurotropic viruses invade the CNS by various routes, including retrograde axonal transport from infected motor and olfactory neurons, infected immune cells that cross the brain endothelium, or by direct infection of cells at the blood–brain barrier (BBB), blood-cerebrospinal fluid barrier, and the meningeal-cerebrospinal fluid barrier (Ludlow et al., 2016). BBB dysfunction appears to be the main route for viral neuroinvasion, and it is preceded by changes in the expression of cytokines, chemokines, cell adhesion molecules (Kim, 2008), and disruption of tight junction (TJ) integrity, as observed in human immunodeficiency virus (HIV) (Pu et al., 2005; Toborek et al., 2005; Strazza et al., 2011; McRae, 2016; Bertrand et al., 2019a), hepatitis C virus (HCV) (Liu et al., 2009; Crema et al., 2015), and West Nile virus (WNV) (Neal, 2014; Suen et al., 2014) infections. Part of this effect is related to high susceptibility of vascular endothelial cells to oxidative and proinflammatory insult (Toborek et al., 1995; Lee et al., 2004), combined with efficient intercellular communication (Cho et al., 2017). HCV and WNV, together with several other major human pathogens, including Japanese encephalitis virus, dengue virus, and Zika virus (ZIKV), are members of the *Flaviviridae* family of viruses. These viruses have significant neuroinvasive characteristics and are regarded as neurotropic (Neal, 2014).

Zika virus is an enveloped, mosquito-borne flavivirus, containing a single-stranded positive-sense RNA genome (Yun and Lee, 2017). Two main lineages, named African and Asian, have been identified so far from the entire genome sequencing data. The African lineage is composed of clusters from Uganda and Nigeria, and the majority of strains were isolated from enzootic vectors, whereas the Asian lineage has been associated with the majority of the human epidemics (Weaver et al., 2016). Compared to other flavivirus, ZIKV is less neuroinvasive, rarely causing meningitis and encephalitis (Carteaux et al., 2016). ZIKV can infect a variety of cells, including neural progenitor cells, causing microcephaly and neurodevelopmental injuries (Tang et al., 2016). Human and animal model studies have observed ZIKV virions and ZIKV nucleic acid in the semen, saliva, tears, urine, eyes, brain, testes, and female genital tract (Miner and Diamond, 2017). ZIKV infects endothelial cells derived from the aorta, brain, and lymphatic and umbilical vessels (Liu et al., 2009, 2016; Richard et al., 2017). In brain microvascular endothelial cells (BMEC), ZIKV infection persists, despite innate antiviral responses, functioning as a viral reservoir capable of spreading the infection into neuronal compartments (Retallack et al., 2016).

In recent years, ZIKV has been recognized as the cause of severe neurological disorders, such as Guillain-Barre syndrome in adults (Oehler et al., 2014; Cao-Lormeau et al., 2016; Watrin et al., 2016) and microcephaly in infants (Brasil et al., 2016a, b; Schuler-Faccini et al., 2016). Unlike other flavivirus, ZIKV can be transmitted via sexual contact (Foy et al., 2011; Musso et al., 2015; Davidson et al., 2016) and possibly by transfusion of whole blood and/or blood products (Barjas-Castro et al., 2016; Motta et al., 2016). Therefore, ZIKV stands as an emerging virus with multiple routes of transmission, capable of crossing the BBB independently of the route of infection. Here, we aimed to characterize the role of the BBB in the onset of ZIKV infection of the CNS, and to characterize possible differences in neurovirulence according to viral strains from the African and Asian lineages, using both *in vivo* and *in vitro* models of ZIKV-infection.

## Materials and Methods

### Cell Culture

C6/36 cells (*Aedes albopictus* clone, mosquito cells) and Vero cells (African green monkey kidney epithelial cells) were obtained from the American Type Culture Collection (ATCC). C6/36 cells (ATCC, CRL-1660) were cultured in Dulbecco’s Modified Eagle Medium (DMEM) containing GlutaMAX (Thermo Fisher Scientific) and supplemented with 10% fetal bovine serum (FBS) (Thermo Fisher Scientific) at 28°C with 5% CO_2_. Vero cells (ATCC CCL-81) were cultured in DMEM containing GlutaMAX (Thermo Fisher Scientific) and supplemented with 10% heat-inactivated fetal bovine serum (FBS) (Thermo Fisher Scientific) at 37°C with 5% CO_2_. Primary human BMECs (Cell Systems, ACBRI 376) were cultured in pre-coated plates with Attachment Factor (Cell Systems) and with Cell Systems Medium, pre-formulated with 10% serum and supplemented with CultureBoost containing animal derived growth factors (Cell Systems), at 37°C with 5% CO_2_.

### Virus Propagation and Tittering

Zika virus strains R103451 (ATCC VR-1848, Honduras 2015, ZIKV-H), PRVABC9 (ATCC VR-1843, Puerto Rico 2015, ZIKV-PR), and MR 766 (ATCC VR-1838, Uganda 1947, ZIKV-U) were obtained from ATCC and propagated in C3/36 mosquito cells at a multiplicity of infection (MOI) of 0.01. Supernatants were collected three to four days post-infection (dpi), clarified by centrifugation at 1,000 × *g* for 5 min, and concentrated using centrifugal filters with 30 kDa molecular weight cut-off (EMDMilipore). Virus was tittered by plaque assay using Vero cells with a 0.7% agarose overlay. Foci of plaques were detected at 3 dpi, following fixation with 10% paraformaldehyde solution and staining with 1% crystal violet.

### Mouse Infection

All animal procedures were approved by the University of Miami Institutional Care and Use Committee (IACUC) in accordance with the National Institutes of Health (NIH) guidelines. Male and female C57BL/6J mice (aged 10–12 weeks) were purchased from Jackson Laboratory and randomly assigned to various ZIKV infection groups. Mice were anesthetized intraperitoneally with a mixture of ketamine (100 mg/kg body weight) and xylazine (5 mg/kg body weight), then injected intravenously via retro-orbital venous sinus with 100 μL of 10^7^ plaques forming units (PFU) of ZIKV-H, ZIKV-PR, ZIKV-U, or vehicle (PBS; mock-infection). After animals were euthanized, whole blood was collected via cardiac puncture, followed by perfusion with normal saline. Brain and spleen tissue were harvested, snap frozen in liquid nitrogen, and stored at –80°C. Whole blood samples were centrifuged at 1,000 × *g* for 10 min to obtain plasma and stored at –80°C.

### Assessment of ZIKV Infection

Total RNA was isolated from 200 μL of plasma or cell culture supernatant using MinElute Virus Spin kit (Qiagen). Total RNA was also isolated from 400 μL of tissue homogenates (spleen and half brain) or 100 μL of cell culture lysates, using RNeasy tissue kit (Qiagen). RT-qPCR of plasma and tissue total RNA samples was performed using primers specific for all ZIKV strains evaluated in the present study (5′-CCG CTG CCC AAC ACA AG-3′ and 5′-CCA CTA ACG TTC TTT TGC AGA CAT-3′), probe (5′-6FAM-AGC CTA CCT TGA CAA GCA GTC AGA CAC TCA A-IABkFQ-3′) (Integrated DNA Technologies), and the qScript XLT 1-Step RT-qPCR ToughMix (Quantabio) reaction mix in a 7500 Real-Time PCR System (Thermo Fisher Scientific). RNA absolute quantification per cell was achieved by fitting to serially diluted standard curves of plasmids containing the target region of ZIKV (10^8^ to 10^1^ viral copies) and the mouse housekeeping gene hemoglobin beta chain complex (HBB) (10^8^ to 10^1^ gene copies), as previously described by our group (Bertrand et al., 2019b). Viral copy numbers less than 10^1^ were considered undetectable and were plotted as zero in the graphs.

### Brain Microvessel Isolation

Mouse brain microvessels were isolated as previously described (Park et al., 2013; Andras et al., 2017). Briefly, brains were homogenized in cold isolation buffer (102 mM NaCl, 4.7 mM KCl, 2.5 mM CaCl_2_, 1.2 mM KH_2_PO_4_, 1.2 mM MgSO_4_, 15 mM HEPES, 25 mM NaHCO_3_, 10 mM glucose, 1 mM Na pyruvate) with freshly added protease inhibitors (Thermo Fisher Scientific) and filtered through a 300 μm nitrocellulose mesh filter (EMDMilipore). Then, 26% dextran (MW, 150 kDa) in isolation buffer was added to the filtered brain homogenate, mixed, and centrifuged at 5,800 × *g* for 20 min at 4°C. The supernatant was removed, pellets were resuspended in isolation buffer, and filtered through a 120 μm nitrocellulose mesh filter (EMDMilipore). Filtered homogenates were then re-pelleted by centrifugation (1,500 × *g*, 10 min, 4°C) and re-resuspended either in 200 μL of radioimmunoprecipitation assay (RIPA) buffer (Thermo Fisher Scientific) supplemented with protease inhibitors for immunoblotting, or in PBS for RNA sequencing.

### Immunoblotting and Immunostaining

Protein fractions from cell culture lysates and isolated brain microvessels were extracted with RIPA buffer supplemented with protease inhibitors and 1% Triton X-100 to inactivate ZIKV. Protein concentrations were measured by Pierce BCA Protein Assay Kit (Thermo Fisher Scientific). The proteins (20 μg/well) were loaded on sodium dodecyl sulfate (SDS) polyacrylamide 4–20% ready gels (BioRad) and electrotransferred to a nitrocellulose membrane using a transfer pack system (BioRad). The blots were probed overnight at 4°C with the following primary antibodies: rabbit anti-ZIKV NS-1 (GeneTex; #GTX133307), rabbit anti-Claudin-5 (Abcam; #ab15106), rabbit anti-ZO-1 (Abcam; #ab96587), and rabbit anti-Occludin (Abcam; #ab167161) (1:1,000) in 5% BSA in TBS-T, overnight at 4°C. Then, the samples were incubated with conjugated secondary anti-rabbit antibody (Licor) (1:20,000) in 5% BSA in TBS-T, for 1 h at room temperature. The blots were washed three times in TBS-T for 5 min after each incubation step. Membranes were imaged in the Licor CLX imaging system and signal quantification was performed using ImageStudio 4.0 software. The GAPDH housekeeping gene was used as a reference.

Immunostaining was performed on primary human BMECs cultured on 8-well chamber slides (Thermo Fisher Scientific). Upon 80% confluency, cells were infected with different strains of ZIKV (ZIKV-H, ZIKV-PR, ZIKV-U, or mock-infected controls) at MOI 0.01 for 48 h. After the incubation period, cells were carefully washed twice with PBS and fixed with 4% paraformaldehyde solution. Fixed cell monolayers were permeabilized with 0.1% Triton-X solution in PBS, followed by blocking with 4% bovine serum albumin (BSA) in PBS for 1 h at room temperature. Chamber slides were incubated overnight at 4°C with rabbit antibodies against the ZIKV NS-1 protein (GeneTex; #GTX133307) (diluted 1:100 in 4% BSA in PBS). Anti-rabbit Alexa-594 secondary antibody (diluted 1:500 in 1% BSA in PBS) (Thermo Fisher Scientific) and Hoechst (diluted 1:2,000 in PBS) (Thermo Fisher Scientific) were used for detection of ZIKV NS-1 protein and cell nuclei, respectively. Imaging was performed on an Olympus FluoView 1200 confocal microscope with a 60× oil immersion lens and analyzed using ImageJ software.

### *In vitro* and *in vivo* Permeability Assays

*In vitro* endothelial permeability assays were performed in 6-well 0.4 μm pore transwell plates (Corning). Primary human BMECs were seeded at 2 × 10^5^ cells per insert in appropriate media, which was changed every 48 h, and transendothelial electrical resistance (TEER) measurements were acquired daily (data not shown). Five days after seeding, cells were inoculated with different strains of ZIKV at MOI 10, diluted in the appropriate media, in quadruplicates. At 2 dpi, media was replaced with Hanks balanced salt solution in both chambers, and fluorescently tagged dextran (FITC-Dextran, Sigma-Aldrich) of 10 kDa or 20 kDa was added to the upper chamber at a final concentration of 0.5 mg/mL. Fluorescent marker translocation was analyzed after 90 min of incubation by transferring 100 μL aliquots from the lower chamber to a 96-well plate and reading fluorescence at 485 nm (Ex) and 525 nm (Em).

*In vivo* BBB permeability assays were performed using sodium fluorescein (NaF) as described before (Zhong et al., 2012; Park et al., 2013, 2016). Briefly, 20 min before euthanasia mice were injected intraperitoneally with 100 μL of 10% sodium fluorescein. Blood was collected via heart puncture, and the mice were transcardially perfused with saline to remove blood from the intravascular compartment. Brains were harvested and thoroughly homogenized in PBS, containing freshly added protease inhibitors, and cleared of debris by centrifugation (1,250 × *g*, 5 min, at 4°C). Proteins were precipitated with 100% trichloroacetic acid (TCA; Sigma-Aldrich) overnight at 4°C. After the incubation period, samples were centrifuged at 10,000 × *g*, 15 min, at 4°C and supernatants were harvested and mixed with 0.05 M sodium tetraborate buffer. A NaF standard curve was prepared by serially diluting 0.01% NaF in saline at a 1:2 ratio, and a total of 12 NaF concentrations were used. NaF fluorescence was measured in a fluorescent plate reader (Ex: 485 nm; Em: 525 nm). Protein levels were measured by the Pierce BCA Protein Assay Kit for normalization of NaF fluorescence.

### Mouse Brain Microvessel Transcriptome

A total of 32 animals were infected with different strains of ZIKV (ZIKV-H, ZIKV-PR, ZIKV-U) or vehicle to characterize the impact of infection on transcriptome of brain microvessels. Each group was composed of 8 animals, 4 males and 4 females. Animals were sacrificed at 2 dpi and whole brain tissue was submitted for microvessel isolation, as described above and elsewhere (Park et al., 2013). Total RNA was isolated using the iPrep PureLink Total RNA kit (Thermo Fisher Scientific). RNA quantitation and quality analyses were performed using Qubit RNA HS Assay kit (Thermo Fisher Scientific) and Agilent RNA 6000 Pico kit (Agilent Technologies). RNA-Seq libraries were prepared using the TruSeq mRNA Library Prep Kit (Illumina), according to the manufacturer’s instructions. To evaluate fragment average and quantify libraries, Agilent 2100 Bioanalyzer, High Sensitivity DNA kit (Agilent Technologies), and a qPCR-based KAPA library quantification kit (KAPA Biosystems) were employed. Libraries were pooled into 20 pM final concentration and submitted to the sequencing reaction using the MiSeq Reagent kit (Illumina) and the MiSeq Sequencing System (Illumina). Reads were trimmed to remove low quality base at the ends and assembled using the CLC Genomics Workbench platform (Qiagen). The total gene hit counts and reads per kilobase million (RPKM) were calculated using the same platform. Gene expression data were compared between groups using the DESeq2 algorithm (Love et al., 2014).

### Statistical Analysis

Experimental treatments were compared pairwise with control treatments using two-way ANOVA, followed by Dunnett’s multiple comparison test of Student’s *t* test with significance value at *p* < 0.05.

## Results

### Different Strains of ZIKV Productively Infect BMEC

**FIGURE 1:**
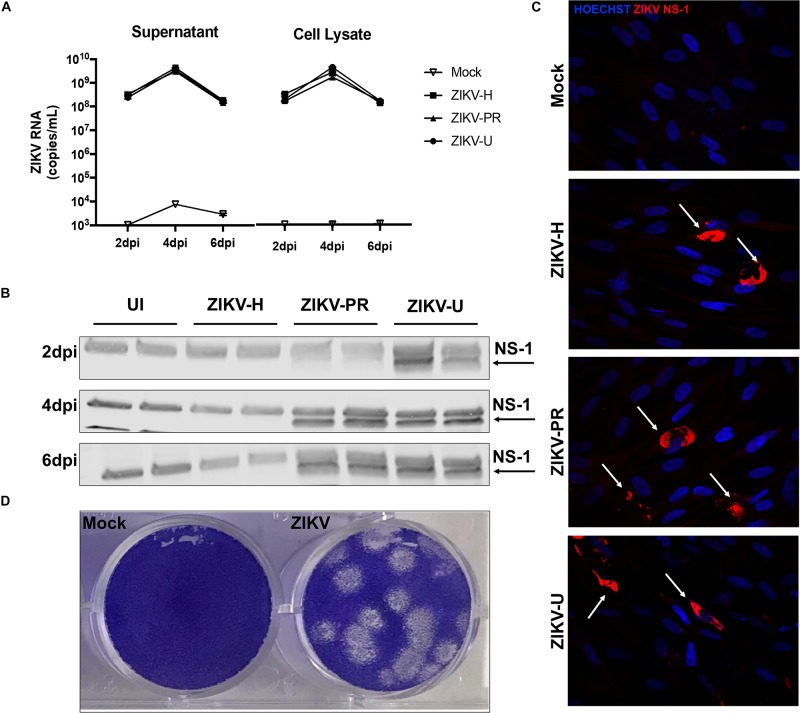
Different strains of ZIKV productively infect brain microvascular endothelial cells (BMECs). Primary human BMECs were infected with different strains of ZIKV: Honduras (ZIKV-H), Puerto Rico (ZIKV-PR), and Uganda (ZIKV-U) at MOI 0.01 for 2, 4, and 6 days. Mock-infected controls (Mock) were treated with virus-free media. **(A)** ZIKV quantification by RT-qPCR, expressed as ZIKV RNA copies per mL in cell culture supernatant (left graph) and cell culture lysates (right graph). **(B)** Detection of ZIKV NS-1 protein expression by immunoblotting in BMEC culture lysates. **(C)** Confocal images of ZIKV-infected BMECs stained for ZIKV NS-1 protein (red; arrows). Cell nuclei were stained with Hoechst (blue). Images are representative of three individual experiments (*n* = 6). **(D)** Representative image of the plaque assay in Vero cells inoculated with BMEC culture supernatant at 2 dpi, showing infectivity of viral particles produced by infected BMECs. Dpi: days post-infection.

We hypothesized that ZIKV, as other neuropathogenic viruses, invades the CNS by disrupting the BBB. Therefore, our first approach was to verify the infectivity of different strains of ZIKV in BMECs, the main component of the BBB. Primary human BMECs were cultured on 6-well plates, and when 80% confluency was reached, cells were infected with ZIKV-H, ZIKV-PR, ZIKV-U at MOI of 0.01, in quadruplicates, or exposed to media only (mock-infected control). The inoculum was left in contact with the cells for 2 h to allow viral adsorption, carefully washed with PBS, and then replaced with fresh uninfected media. Cell culture supernatants and cell lysates were collected at 2, 4, and 6 dpi, and submitted to RNA extraction. ZIKV levels were assessed by RT-qPCR in cell culture supernatants and BMEC lysates (Figure 1A, left and right panels, respectively). At 2 dpi, 10^8^ ZIKV RNA copies/mL were detected in both supernatants and cell lysates. The viral burden reached a peak at 4 dpi (approximately 10^10^ ZIKV RNA copies/mL in the supernatants and cell lysates), and at 6 dpi viral load decreased to the levels found at 2 dpi, the effect that was accompanied by cell detachment as assessed by microscopy, suggesting loss of cell viability (data not shown).

Productive infection was also assessed by expression of viral proteins. ZIKV-infected BMEC lysates were submitted to immunoblotting for NS-1 ZIKV protein (Figure 1B). NS-1 was detected as early as 1 (data not shown) and 2 dpi in ZIKV-U-infected BMEC. At 4 and 6 dpi, NS-1 expression was observed in ZIKV-PR and ZIKV-U-infected cells; however, no NS-1 protein was detected in ZIKV-H-infected cells (Figure 1B). On the other hand, ZIKV infection of BMECs by immunofluorescence was detected in cultures exposed to all ZIKV strains employed in this study. In these experiments, BMECs were seeded on 8-well chamber slides, infected at 80% confluency and MOI 0.01, and stained for ZIKV NS1 protein (red) and DAPI (blue) at 2 dpi (Figure 1C, arrows).

We next demonstrated that BMECs are not only permissive to productive ZIKV infection, but these cells also produce infectious viral particles. Supernatants from ZIKV-H-infected BMECs were collected at 2 dpi and used to infect naïve Vero cells. Infection was detected by a plaque assay using an agarose overlay and crystal violet staining (Figure 1D).

### Impact of ZIKV Infection on Endothelial Permeability and TJ Protein Expression *in vitro*

**FIGURE 2:**
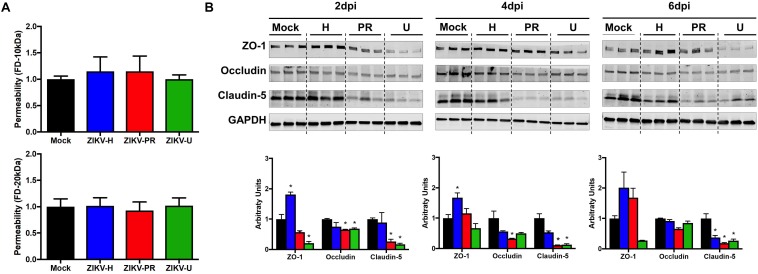
ZIKV infection impact on endothelial cell permeability and tight junction protein expression. **(A)** Using a transwell system, primary human brain microvascular endothelial cells (BMECs) were infected with different strains of ZIKV: Honduras (ZIKV-H), Puerto Rico (ZIKV-PR), and Uganda (ZIKV-U) at MOI 10. Mock-infected controls (Mock) were treated with virus-free media. At 2 dpi, culture media in the apical compartment of the transwell system was replaced with medium containing fluorescently tagged dextran (FD) of 20 kDa (upper panel) and 10 kDa (lower panel). Basolateral levels of FD were assayed 90 min post-addition of FD-dextran. Data are mean ± SEM, expressed as fold increase over mock-infected control; three independent experiments, each with *n* = 6. **(B)** Expression of ZO-1, occludin, and claudin-5 was assessed by immunoblotting at 2, 4, and 6 dpi in primary human BMECs infected with ZIKV-H (blue), ZIKV-PR (red), and ZIKV-U (green) at MOI 0.01. Mock-infected controls (Mock; black) were treated with virus-free media. Presented blots are cropped from the originals. Data are mean ± SEM expressed as fold increase over mock-infected control. ^∗^*p* < 0.05 vs. mock-infected.

To evaluate the impact of ZIKV infection on endothelial permeability, human primary BMECs were grown to confluence on transwell inserts. Preliminary permeability experiments (data not shown) were performed with BMECs infected with ZIKV-H at MOI 0.01 and 1; however, no alteration of BMEC barrier integrity was detected; therefore, the subsequent experiments were performed at MOI 10 to ensure that all cultured cells were infected. Control cultures were exposed to medium only. Then, media in the apical compartment of the transwell system was replaced with medium containing fluorescently tagged dextran of either 20 kDa or 10 kDa (Figure 2A, upper and lower panels, respectively). After 90-min incubation, fluorescence intensity in the basal compartment was detected using a plate reader. Infection with all ZIKV strains employed in the current study did not induce significant differences in endothelial permeability.

Intriguingly, ZIKV infection caused alterations of the levels of TJ proteins. Cells were infected with ZIKV-H, ZIKV-PR, ZIKV-U, or mock-infected (media only) at MOI 0.01 and lysed in RIPA buffer at 2, 4, and 6 dpi (Figure 2B). The expression levels of ZO-1, occludin, and claudin-5, three major TJ proteins involved in maintaining endothelial permeability, were analyzed by immunoblotting. A significant reduction in claudin-5 levels was observed in cells infected with ZIKV-PR and ZIKV-U throughout the infection time points analyzed. In addition, a significant downregulation of claudin-5 in ZIKV-H-infected cells compared to mock-infected controls was observed at 4 dpi and sustained at 6 dpi. On the other hand, ZIKV-H-infected cells presented significantly increased expression of ZO-1 at 2, 4, and 6 dpi. Interestingly, at 2 dpi, expression of ZO-1 and occludin was significantly decreased in ZIKV-PR and ZIKV-U-infected cells, compared to mock-infected controls. Downregulation of occludin persisted at 4 and 6 dpi in ZIKV-PR-infected cells (Figure 2B).

### ZIKV Infection of Immunocompetent Mice

**FIGURE 3:**
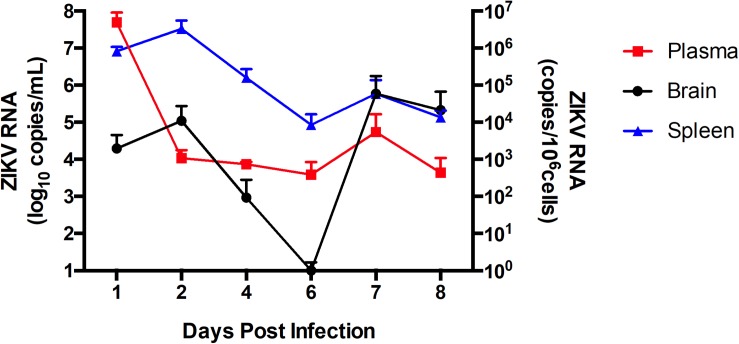
ZIKV infection kinetics in immunocompetent mice. C57BL/6J mice were infected intravenously with 10^7^ PFU of ZIKV-PR or vehicle and euthanized at 1 dpi (*n* = 4), 2 dpi (*n* = 4), 4 dpi (*n* = 4), 6 dpi (*n* = 4), and 8 dpi (*n* = 4). ZIKV RNA quantification was assessed in plasma (in red; expressed as RNA copies/mL of blood), spleen (in blue; expressed as RNA copies/10^6^ cells) and brain homogenates (in black; expressed as RNA copies/10^6^ cells) by qPCR. Data are mean ± SEM.

Following the *in vitro* analyses, we next evaluated the impact of ZIKV infection on BBB permeability and TJ protein expression *in vivo*. In order to assess the kinetics of ZIKV infection, immunocompetent C57BL/6J male mice were infected with 10^7^ PFU of ZIKV-PR or vehicle and euthanized at 1 dpi (*n* = 4), 2 dpi (*n* = 4), 4 dpi (*n* = 4), 6 dpi (*n* = 4), and 8 dpi (*n* = 4). Systemic infection was verified by the presence of ZIKV RNA in spleen tissue homogenates and plasma samples by ZIKV-specific RT-qPCR. In brain tissue homogenates, ZIKV RNA levels peaked at 2 dpi (Figure 3). Therefore, all remaining animal experiments were performed at this time point.

**FIGURE 4:**
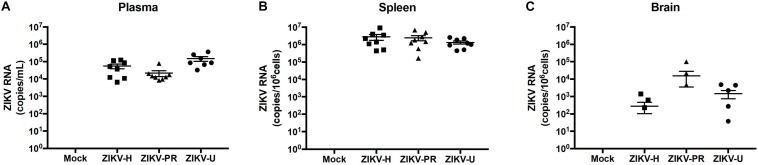
Different strains of ZIKV infect immunocompetent mice. C57BL/6J mice were infected intravenously with 10^7^ PFU of ZIKV-H, ZIKV-PR, and ZIKV-U, and euthanized after 48 h. Mock-infected controls were injected intravenously with PBS. ZIKV RNA quantification was assessed in plasma (**A**; expressed as RNA copies/mL of blood), spleen (**B**; expressed as RNA copies/10^6^ cells) and brain homogenates (**C**; expressed as RNA copies/10^6^ cells) by qPCR. Viral copy numbers less than 10^1^ were considered undetectable and were plotted as equal to zero. ZIKV RNA was detected for all animals in plasma and spleen homogenates (*n* = 8 per group). Brain homogenates were positive for ZIKV RNA in 3/8, 3/8, and 5/8 animals infected with ZIKV-H, ZIKV-PR, and ZIKV-U, respectively. Data are mean ± SEM.

A total of 8 animals (4 males and 4 females) per group (groups: ZIKV-H, ZIKV-PR, ZIKV-U, vehicle) were infected intravenously with 10^7^ PFU of ZIKV and euthanized at 2 dpi. Plasma, spleen and brain tissues were collected and processed for RNA extraction, followed by ZIKV detection by RT-qPCR. Figure 4 depicts the RNA quantification for each tissue analyzed and for individual ZIKV strains. No differences were observed between male and female genders. Therefore, the results from male and female mice were combined.

Zika virus RNA levels in plasma samples ranged from 6.5 × 10^3^ to 1.2 × 10^5^ copies/mL in ZIKV-H-infected animals, from 8.3 × 10^3^ to 0.8 × 10^5^ copies/mL in ZIKV-PR-infected animals, and from 0.3 × 10^3^ to 3.7 × 10^5^ copies/mL in ZIKV-U-infected animals (Figure 4A). One female mouse infected with ZIKV-U had undetectable levels of ZIKV RNA in the plasma, and this animal was excluded from further analyzes.

Spleen levels of ZIKV RNA were the highest compared to the other tissues analyzed (Figure 4B), with the mean value 2.8 × 10^6^ copies/10^6^ cells for ZIKV-H-infected animals, 2.5 × 10^6^ copies/10^6^ cells for ZIKV-PR-infected animals, and 1.3 × 10^6^ copies/10^6^ cells for ZIKV-U-infected animals.

Brain infection was detected in 3 out of 6 mice infected with ZIKV-H or with ZIKV-PR, and in 5 out of 6 mice infected with ZIKV-U. Mean levels of ZIKV RNA showed tendency to be slightly lower in ZIKV-H-infected animals (2.8 × 10^2^ copies/10^6^ cells) compared to ZIKV-PR (1.6 × 10^4^ copies/10^6^ cells) and ZIKV-U (1.5 × 10^3^ copies/10^6^ cells); however, the differences were not significant (Figure 4C) (ANOVA; *F* = 1.537; *p* = 0.2268). These data suggest that immunocompetent mice are a feasible model to study ZIKV infection, including neuroinfection.

### Brain Infection by ZIKV-H Correlates With BBB Disruption

**FIGURE 5:**
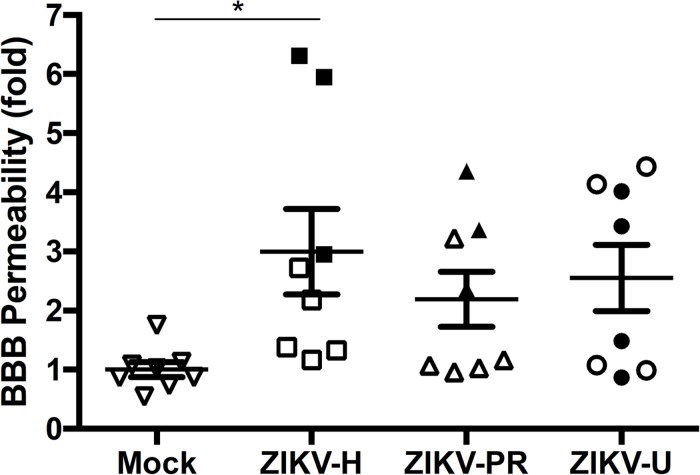
Impact of ZIKV infection in BBB permeability *in vivo*. C57BL/6J mice were infected intravenously with 10^7^ PFU of ZIKV-H, ZIKV-PR, and ZIKV-U, and euthanized after 48 h. Mock-infected controls were injected intravenously with PBS. Approximately 20 min before euthanasia, animals were treated intraperitoneally with 10% sodium fluorescein (NaF, 376 Da). Translocation of NaF from plasma into the brain parenchyma was used as the indicator of BBB integrity. Closed symbols indicate animals with ZIKV-infected brains; open symbols indicate animals with no brain infection. Data are mean ± SEM, expressed as fold change compared to mock-infected control, *n* = 8 per group. ^∗^*p* = 0.0078, vs. mock-infected control.

Our next series of experiments focused on the impact of ZIKV infection on BBB permeability as assessed by the NaF extravasation assay. The same animals that were used to evaluate ZIKV-induced brain infection in Figure 3 were injected intraperitoneally with 10% NaF, the dye was allowed to circulate for 20 min, and NaF extravasation into the brain parenchyma was measured as previously established in our laboratory (Park et al., 2013). Infection with ZIKV-H induced significant increase in the mean levels of NaF fluorescence in the brain, compared to mock-infected controls (Figure 5). While the mean levels of NaF fluorescence in the brains of ZIKV-PR and ZIKV-U-infected animals were approximately 2-fold higher compared to mock-infected controls, no statistical significance was detected due to high in-group variability (Figure 5).

Results presented in Figure 3 indicated that 11/24 (45.8%) mice subjected to ZIKV infection developed brain infection and/or disruption of the BBB. Because ZIKV may infect the brain as the result of penetration via the BBB, we attempted to analyze if these changes are associated. Indeed, alterations of BBB permeability were detected in all mice that developed brain infection with ZIKV-H (Figure 5). However, ZIKV-PR and ZIKV-U-infected mice presented a less evident association, suggesting strain-specific effects for ZIKV-H infection.

### Impact of ZIKV Infection on the Expression of TJ Proteins *in vivo*

**FIGURE 6:**
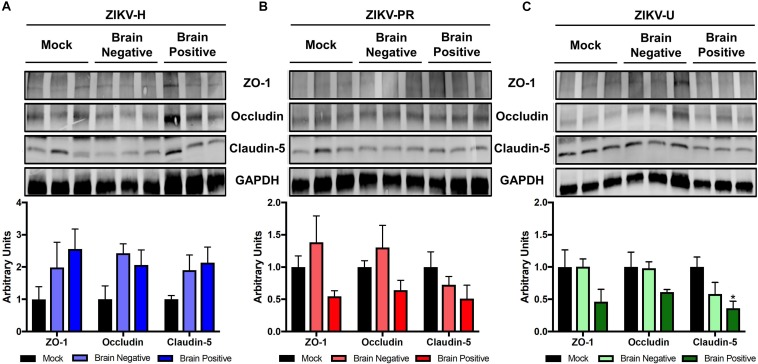
ZIKV infection impact on tight junction proteins expression *in vivo*. Brain microvessels were isolated post-mortem from ZIKV-infected immunocompetent mice at 2 dpi. Expression of ZO-1, occludin, and claudin-5 were assessed by immunoblotting in mock-infected and ZIKV-H **(A)**, ZIKV-PR **(B)**, and ZIKV-U **(C)** infected animals. ZIKV-infected animals were divided according to viral detection in the brain, according to RT-qPCR data (Figure 4) (Brain negative: ZIKV-infected mice with brain RT-qPCR negative; Brain positive: ZIKV-infected mice with brain RT-qPCR positive). GAPDH was used as an internal control. Presented blots are cropped from the originals. Data are mean ± SEM expressed as fold increase over mock-infected control. ^∗^*p* < 0.05 vs. not infected (ANOVA + Dunnett).

Using the microvessels isolated from the brains of mice analyzed in Figures 3, 4, we evaluated the levels of claudin-5, occludin, and ZO-1 by immunoblotting. Mice were grouped according to the detection of ZIKV RNA in their brain, and the analyses were performed separately on infected and mock-infected brains at 2 dpi (Figure 6). Animals infected with ZIKV-H exhibited a trend toward increased levels of TJ proteins in both infected and mock-infected brains as compared to mock-infected controls. ZIKV-PR-infected mice presented a trend toward decreased TJ protein levels in the infected brains, but not in the mock-infected brains. A similar trend in decreased TJ protein expression was observed in ZIKV-U infected brains, with claudin-5 expression reaching statistically significant values (Figure 6).

### Impact of ZIKV Infection on Transcriptome of Brain Microvessels

Our studies on the impact of ZIKV infection on brain microvasculature and the BBB were completed by evaluating the transcriptome profile of brain microvessels by high-throughput RNA sequencing. Eight animals (4 males and 4 females) per group were infected with ZIKV-H, ZIKV-PR, and ZIKV-U for 48 h. Mock-infected animals were included as negative controls. As illustrated in Figure 3, ZIKV titers in the brain show biphasic response, with the first peak at 2 dpi, followed by the second peak at 7 dpi. Protein expression profiles and the transcriptome analysis were performed during the first peak, i.e., at 2 dpi. Whole brain tissue samples were homogenized and microvessels were isolated and submitted to RNA extraction, followed by sequencing, as described in section “Materials and Methods”.

**FIGURE 7:**
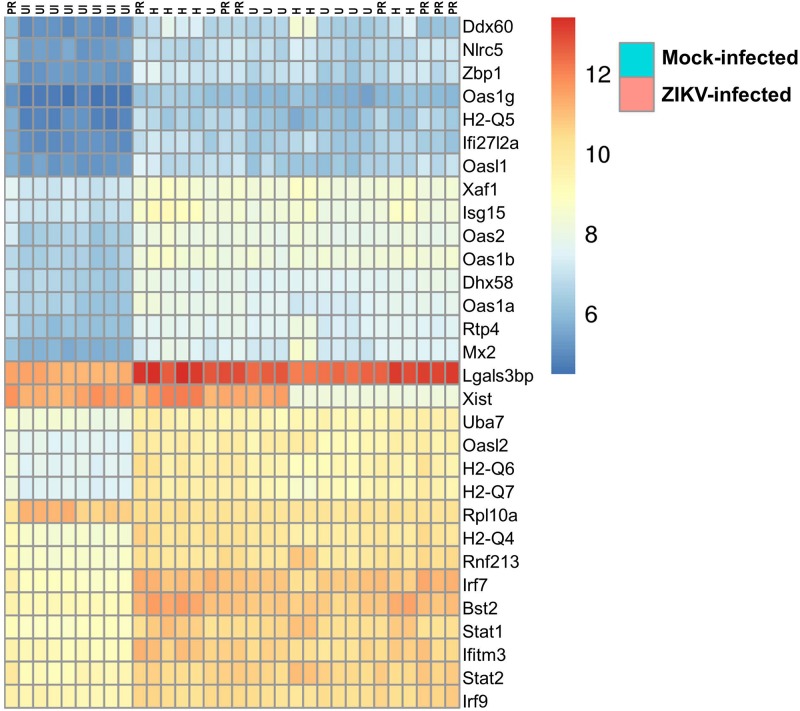
Transcriptome of ZIKV-infected mice. Differentially expressed genes bi-clustering heat map of the top 30 genes sorted by adjusted *p*-values, between mock-infected and ZIKV-infected animals. *n* = 8, per group.

Figure 7 illustrates differentially expressed genes bi-clustering heat map of the top 30 genes sorted by adjusted *p*-values, between mock-infected and ZIKV-infected animals. In addition, Table 1 depicts the top 30 upregulated (in blue) and downregulated (in red) genes according to ZIKV strain and compared to mock-infected controls. Samples are grouped according to the similarities in gene expression.

**TABLE 1:** Mouse brain microvessel transcriptome.

**ZIKV-H**			**ZIKV-PR**			**ZIKV-U**		
		
**Gene**	**Fold change**	**Adj. *p*-value**	**Gene**	**Fold change**	**Adj. *p*-value**	**Gene**	**Fold change**	**Adj**.***p*-value**
Ifi213	6.5	4.7E-14	Ppp2r2cos	5.8	1.2E-12	Eif2s3y	5.5	1.5E-04
Uty	5.9	1.6E-03	Gm29650	5.5	3.8E-03	Uty	5.4	1.6E-03
Ifit1	5.8	1.1E-26	Kdm5d	5.4	2.8E-04	Kdm5d	4.8	9.6E-03
Ddx60	5.7	1.6E-47	Uty	5.0	1.6E-03	Ddx3y	4.6	2.3E-03
Ddx3y	5.6	7.4E-05	Ddx3y	4.7	3.0E-05	Gm14429	4.2	9.8E-16
Gbp6	5.5	3.5E-10	Eif2s3y	4.5	1.1E-02	Gm42943	4.2	4.0E-11
Eif2s3y	5.4	1.1E-02	H2-Q6	4.4	2.0E-44	Ifit1	4.1	6.4E-23
Phf11d	5.4	5.2E-18	Zbp1	4.4	4.1E-36	Irf7	4.1	3.0E-233
Ifi44	5.3	6.2E-65	Irf7	4.3	9.2E-56	Ddx60	3.9	2.2E-51
Kdm5d	5.0	9.9E-03	H2-Q5	4.2	2.6E-32	Oas1b	3.9	6.6E-78
Ifi209	5.0	1.3E-07	H2-Q7	4.2	5.1E-45	Zbp1	3.8	2.2E-42
Gm43196	4.8	1.1E-11	Phf11d	4.1	7.4E-12	H2-Q6	3.8	3.9E-87
Mx1	4.8	1.3E-35	Oasl2	4.1	6.4E-45	Oasl2	3.8	2.7E-93
Ifi204	4.7	1.1E-11	Gm42943	4.1	6.1E-12	Oas3	3.7	5.8E-13
Zbp1	4.7	3.3E-66	Oas3	4.0	1.0E-12	H2-Q7	3.7	1.5E-99
Mx2	4.6	7.0E-65	Gm13833	4.0	2.9E-15	H2-Q5	3.4	4.5E-30
Trim30b	4.5	2.2E-09	9330175E14Rik	4.0	5.2E-12	Gm14411	3.4	2.4E-12
Gbp3	4.5	9.5E-13	Gm14429	3.9	2.2E-14	Gm28177	3.4	5.1E-25
Ifit3	4.3	2.5E-40	Gm16105	3.8	2.6E-31	Mx1	3.4	1.5E-24
Oasl2	4.3	8.9E-156	Oas1b	3.8	9.3E-32	Mx2	3.3	3.7E-79
Oas1b	4.2	4.4E-97	Nlrc5	3.8	9.7E-47	Oas1g	3.3	5.3E-32
Ifi27l2a	4.2	3.5E-66	Ddx60	3.7	2.2E-30	Ifi27l2a	3.3	1.5E-44
Phf11b	4.1	1.9E-09	Mx2	3.7	2.7E-38	Gm44802	3.2	1.1E-13
9330175E14Rik	4.1	2.2E-14	Mx1	3.6	6.5E-23	Nlrc5	3.2	1.3E-46
Tgtp2	4.1	8.4E-08	Gbp3	3.6	8.1E-27	Bst2	3.1	1.0E-200
Oas1g	4.1	2.7E-50	Oasl1	3.6	6.1E-26	Gm15813	3.1	1.7E-09
H2-Q6	3.9	5.8E-49	Ifi27l2a	3.6	6.5E-28	Oas2	3.1	4.1E-59
Trim30a	3.9	4.6E-36	Ifit1	3.6	2.8E-31	Gbp3	3.1	5.4E-16
Bst2	3.9	1.0E-122	Oas1g	3.6	2.2E-25	Ifi44	3.0	2.9E-20
Ifit3b	3.9	6.7E-22	4921534H16Rik	3.5	7.7E-11	Gm26869	3.0	1.8E-21
Rps15-ps2	−1.8	2.7E-04	Gm43018	−2.5	6.3E-19	Gm10736	−1.9	1.4E-15
Map3k10	−1.8	1.4E-13	Gm12987	−2.5	2.0E-09	Mgl2	−1.9	4.3E-10
Gp9	−1.8	6.4E-07	mt-Ta	−2.6	2.5E-46	Gm22739	−1.9	8.0E-03
Git1	−1.8	6.7E-13	Gm37165	−2.6	1.8E-14	Gm47271	−1.9	1.5E-12
Fth1	−1.8	2.5E-28	Rps11-ps1	−2.6	2.5E-31	Gm4149	−1.9	1.5E-11
Kcnab2	−1.8	5.9E-14	Gm26638	−2.6	1.8E-20	Rpl37a	−1.9	1.6E-24
Ttbk1	−1.9	6.8E-19	Gm15700	−2.6	3.0E-09	mt-Ta	−1.9	1.7E-15
Ddn	−1.9	1.7E-21	2810432F15Rik	−2.6	9.0E-15	Tsix	−1.9	1.6E-04
Brsk1	−1.9	3.2E-18	Gm42443	−2.6	3.3E-17	Gm12987	−1.9	4.1E-07
mt-Tq	−1.9	6.8E-10	2900002M20Rik	−2.7	6.9E-16	Rps27rt	−1.9	9.0E-10
Scrt1	−1.9	6.1E-16	Gm17981	−2.7	1.2E-23	Gm10123	−2.0	3.8E-22
Gkn3	−2.0	6.3E-12	Gm23127	−2.7	5.7E-13	Gm24336	−2.0	7.5E-09
Ccdc85c	−2.0	1.6E-14	Gm38083	−2.7	4.3E-15	Rpl3-ps1	−2.0	2.2E-15
Neurl1a	−2.0	5.1E-15	Gm24927	−2.8	9.0E-17	Omp	−2.0	2.0E-02
Mgl2	−2.0	1.6E-10	Gm26692	−2.8	4.1E-11	Gm6204	−2.0	1.0E-08
Alas2	−2.0	8.7E-11	4930528J11Rik	−2.8	2.9E-09	Gm17409	−2.0	9.2E-09
Dlgap3	−2.0	6.0E-18	Gm24492	−2.9	1.2E-13	Rpl10a-ps1	−2.1	3.6E-13
Mbp	−2.0	1.5E-26	Gm47271	−2.9	4.6E-22	Rps10-ps1	−2.1	1.5E-09
Tmcc2	−2.1	6.1E-17	Gm24406	−2.9	4.9E-16	Gm7536	−2.1	6.4E-11
Plekhb1	−2.1	2.3E-29	Gm37436	−2.9	2.2E-09	Rps28	−2.2	4.5E-22
Ppp1r9b	−2.2	1.7E-18	Gm43884	−3.0	4.6E-07	Alas2	−2.2	1.2E-29
Rasl10b	−2.2	4.0E-16	Hba-a1	−3.1	3.7E-07	Gm5805	−2.2	1.4E-12
Gm6204	−2.2	5.2E-08	Tsix	−3.1	2.6E-11	Slc4a1	−2.3	1.2E-07
Phospho1	−2.3	4.1E-10	Hba-a2	−3.1	7.0E-07	Gm15700	−2.3	7.5E-08
Omp	−2.3	9.5E-03	Gm22739	−3.1	2.0E-05	Mageb16-ps1	−2.8	9.8E-15
Hbb-bs	−2.8	4.1E-13	Gm37211	−3.2	4.8E-27	Hba-a1	−3.8	2.1E-50
Hba-a1	−2.8	2.2E-12	Gm26129	−3.3	1.4E-11	Hba-a2	−3.8	2.6E-52
Hba-a2	−2.8	2.4E-14	Mageb16-ps1	−3.5	4.2E-05	Xist	−4.3	4.7E-05
Xist	−3.8	8.0E-04	Gm17409	−3.9	3.7E-16	Hbb-bs	−4.5	6.2E-89
Hbb-bt	−3.9	3.0E-13	Xist	−4.4	4.3E-05	Hbb-bt	−5.2	7.3E-56

*The top 30 upregulated (in blue) and downregulated (in red) genes according to ZIKV strain and compared to mock-infected controls.*

The transcriptome of brain microvessels (Table 1) from animals infected with ZIKV-H, ZIKV-PR, and ZIKV-U was quite uniform, except for the Xist gene that was downregulated in male animals, compared to females (*p* = 2.4 × 10^–26^). The Xist gene ensures X-chromosome inactivation in female placental mammals, and therefore its expression is expected to be low in male animals. Gene expression related to BBB function was not significantly changed in microvessels isolated from ZIKV-infected animals as compared to mock-infected controls. However, the analysis revealed upregulation of genes involved in immune responses against viral infection, such as Ddx60 (*p* = 1.2 × 10^–24^), Dhx58 (*p* = 8.8 × 10^–45^) and Nlrc5 (*p* = 5.4 × 10^–45^). In addition, IFN-related immune response genes, such as Zbp1 (*p* = 6.8 × 10^–44^) and genes of the Oas (Oasl2, *p* = 8.1 × 10^–72^; Oas1b, *p* = 5.9 × 10^–59^; Oas3, *p* = 5.3 × 10^–13^; Oas1g, *p* = 3.0 × 10^–34^; OasL1, *p* = 1.7 × 10^–25^; Oas2, *p* = 1.0 × 10^–59^; Oas1a, *p* = 9.3 × 10^–38^), H2-Q (H2-Q4, *p* = 3.5 × 10^–53^; H2-Q5, *p* = 4.1 × 10^–30^; H2-Q6, *p* = 5.2 × 10^–55^; H2-Q7, *p* = 1.6 × 10^–54^), Irf (Irf7, *p* = 9.8 × 10^–86^; Irf9, *p* = 1.0 × 10^–33^), and Stat (Stat1, *p* = 1.1 × 10^–46^; Stat2, *p* = 5.9 × 10^–75^) families were upregulated in microvessels of ZIKV-infected animals. Interestingly, the gene Rnf213 was significantly upregulated in ZIKV-infected animals (*p* = 3.9 × 10^–46^). Rnf213 encodes a type Zn-finger protein involved in mediating protein-protein interactions and has been reported as a susceptibility gene for Moyamoya disease, a vascular disorder of intracranial arteries (Liao et al., 2017). In addition, expression of the gene encoding the cellular glycoprotein galectin 3 binding protein (Lgals3bp) was significantly upregulated in ZIKV-infected animals, compared to mock-infected animals (*p* = 3.0 × 10^–28^). The expression of Lgals3bp is upregulated in the context of HIV-1 and HCV infections, and its expression is associated with upregulation of IL-2, IL-6, granulocyte-macrophage colony-stimulating factor (GM-CSF), and TNF-α, contributing to an antiviral state (Lodermeyer et al., 2018).

Regarding the mostly downregulated genes, ribosomal protein encoding genes were significantly downregulated in ZIKV-infected animals, compared to mock-infected animals (Rpl3, *p* = 4.1 × 10^–19^; Rpl19, *p* = 1.7 × 10^–12^; Rpl27a, *p* = 1.7 × 10^–19^; Rpl37a, *p* = 5.3 × 10^–22^; Rps10, *p* = 1.2 × 10^–10^; Rps14, *p* = 4.8 × 10^–12^; Rps27rt, *p* = 7.5 × 10^–7^; Rps28, *p* = 3.1 × 10^–20^). Interestingly, one gene related to neurogenesis signaling pathway and one gene related to cell differentiation signaling pathway (Omp and Plekhb1, respectively) were significantly downregulated in ZIKV-infected animals, as compared to mock-infected animals (*p* = 0.0005 and *p* = 9.5 × 10^–23^, respectively).

## Discussion

The present study aimed to identify the mechanisms by which ZIKV crosses the BBB to infect CNS cells, a condition that has been linked to microcephaly in infants and Guillain-Barré syndrome in adults. Here we used an animal model of ZIKV infection of immunocompetent adult mice to verify possible damages in BBB integrity in the adult population. We chose to analyze different strains of ZIKV in order to verify possible differences in neurovirulence, and therefore in neuropathogenesis.

The viral strains responsible for the recent outbreaks in the Americas, leading to an alarmingly high number of cases of microcephaly, were phylogenetically clustered to Asian strains of ZIKV, previously associated with Guillain-Barré syndrome and microcephaly (Lessler et al., 2016). In Asia, on the other hand, the outbreaks emerged during the 2000 decade, as a consequent spread of the ZIKV burden from Africa (Duffy et al., 2009). African strains of ZIKV have not been associated with human neuropathogenesis and the disease remains predominantly in the sylvatic cycle (Lessler et al., 2016). Therefore, we analyzed the impact of three different strains of ZIKV in BBB integrity: the Honduras and Puerto Rico strains, representing the Asian lineage; and the Uganda strain representing the African lineage.

Our data show that ZIKV infects and replicates in BMECs, the main component of the BBB, independent of viral strain. Moreover, we found no differences in infection and replication efficiency among different strains in these cells, as shown by ZIKV RNA quantification from both cell culture supernatants and cell lysates (Figure 1A). In general, these results are in accordance with previous reports showing that different strains of ZIKV, both of the African and Asian lineages, productively infect and spread in primary (Mladinich et al., 2017; Papa et al., 2017) and cell line cultures of BMECs (Bramley et al., 2017). In contrast, the Asian strains of ZIKV were demonstrated to have faster replication kinetics in human umbilical vein endothelial cells compared to African strains (Liu et al., 2016).

Next, we sought to determine if *in vitro* infection of BMEC was accompanied by alterations in endothelial permeability. *In vitro* endothelial integrity studies were performed using between 10^3^ or 10^6^ copies of ZIKV at MOI 0.01 or 10, respectively. In addition, 10^7^ PFU of ZIKV were employed for *in vivo* BBB permeability assays. We did not observe any disruption of the endothelial barrier function as determined by FITC-dextran flux after ZIKV-U, ZIKV-PR, or ZIKV-H infection, even at MOI of 10 (Figure 2A). Nevertheless, we did not perform Electronic Cell-Substrate Impedance Sensing (ECIS) studies that allow real-time measurements of TEER for detection of short-lived permeability changes. In addition, permeability assessments were performed only at 2 dpi, when the changes in TJ protein expression were the most pronounced but were not continued at later stages of infection. These factors constitute limiting aspects of our *in vitro* ZIKV model. Similar lack of endothelial permeability changes was reported for the ZIKV-U and a ZIKV strain from Brazil (PE243) (Papa et al., 2017). These strains also productively infected primary human BMECs but without enhancing endothelial permeability. One caveat of that report was that endothelial permeability was assessed by the translocation of FITC-tagged BSA (Papa et al., 2017), i.e., a method suitable to detect only very substantial permeability changes. In our study, we used different molecular weight FITC-tagged dextran molecules (10 kDa and 20 kDa) that were smaller than BSA (67 kDa). Thus, our assay was more sensitive, strengthening the conclusion that ZIKV is able to reach the CNS without disrupting the BBB. Instead, invasion of the CNS by ZIKV might be associated with endothelial transcytosis (Papa et al., 2017).

Infections of BMECs with other neurotropic flaviviruses, such as tick-borne encephalitis virus (Palus et al., 2017) and West Nile virus (Roe et al., 2012) were also reported as not resulting in disruption of endothelial barrier function. On the other hand, WNV-induced alterations of the BBB *in vivo* were shown to be transient and regulated by the interaction of the host with viral factors, such as pathogen-associated molecular patterns (PAMPs; e.g., viral RNA) (Daniels et al., 2014; Cain et al., 2017). Recently, Puerta-Guardo et al. (2019) described *in vitro* endothelial cell permeability alterations induced by NS1 proteins of different flaviviruses, including ZIKV. Our *in vivo* studies revealed that significant changes in BBB permeability for NaF (molecular weight 376 Da) occurred only among animals infected with the Honduras strain. In addition, a clear trend between the presence of the neuroinfection and increased BBB permeability was observed in mice infected with ZIKV-H but not ZIKV-U or ZIKV-PR, indicating strain-specific effects (Figure 5). The paradox between the *in vitro* and *in vivo* permeability results may be explained by the limitations of measurements of endothelial permeability with fluorescent dextran polymers. Another possibility may be lack in our *in vitro* model other components of neurovascular unit, such as astrocytic foot processes and contributing microglial cells. Indeed, astrocytes are a critical component of the BBB microenvironment, and secreted products from activated microglial cells contribute to the infectious process (Nau et al., 2014).

Interestingly, when we evaluated the expression of TJ proteins after ZIKV infection, significant changes starting at 2 dpi, and at a physiologically relevant dose (MOI 0.01) were observed (Figure 2B). Expression of claudin-5 and occludin were significantly downregulated in ZIKV-PR and ZIKV-U infected BMECs, whereas ZO-1 expression was upregulated. Changes in TJ protein expression are frequently accompanied by disruption of endothelial integrity, as demonstrated for other neurotropic flaviviruses, such as Japanese encephalitis virus (Kim et al., 2016) and dengue virus (Velandia-Romero et al., 2016). However, the role of occludin in maintaining barrier function is not clearly defined as occludin-deficient mice form paracellular barriers and do not exhibit defects in epidermal, respiratory, or bladder TJ functions (Saitou et al., 2000; Schulzke et al., 2005). While downregulation of claudin-5 has been very strongly associated with an increase in transendothelial permeability (Nitta et al., 2003; Andras et al., 2005; Argaw et al., 2009), it is possible that upregulation of ZO-1 can maintain functional barrier properties by affecting other claudins or other transmembrane TJ proteins.

The *in vivo* analyses of TJ protein expression in ZIKV-infected brains agree with *in vitro* data. In ZIKV-H-infected animals, all TJ proteins analyzed tended to be upregulated. In ZIKV-PR infected animals, a tendency in the upregulation of ZO-1 and occludin expression was evident in mock-infected brains; however, a reverse trend was observed in infected brains. Regarding infection with ZIKV-U stains, TJ proteins were downregulated in infected brains, achieving statistical significance only for claudin-5. Overall, the pattern of the observed changes suggests specific responses from the BBB during neuroinfection by individual strains of ZIKV. However, the kinetics of TJ alterations may depend not only on different viral strains but be individually tuned for individual TJ proteins. It also should be noted that modulation of the BBB permeability is frequently regulated by transient phosphorylation/dephosphorylation of TJ proteins and not their overall expression levels (Siddiqui et al., 2015). Thus, studies on transient changes in TJ phosphorylation status may consist an important future direction of research on the BBB involvement in ZIKV entry into the CNS.

After the recent Zika epidemic, the development of animal models to study the virus pathogenesis has been a research priority. There are approximately 20 mouse models of ZIKV infection published in the literature (Morrison and Diamond, 2017); however, only a few studies were successful in using immunocompetent adult mice (Larocca et al., 2016; Ma et al., 2016; Yockey et al., 2016). Among these studies, ZIKV was detected in blood (Larocca et al., 2016), testes (Ma et al., 2016), and vaginal tissues (Yockey et al., 2016), correlating with the inoculation route (intravenous, intratesticular, and intravaginal, respectively). To our knowledge, our study is the first to show an immunocompetent adult mouse model of systemic and brain infection (Figure 4). Our model could serve as a stepping stone for the development of more robust animal models associated with ZIKV pathogenesis in adults, such as Guillain-Barré syndrome (Morrison and Diamond, 2017). It is well-established that ZIKV infects neural progenitor cells, mature neurons, astrocytes, and brain endothelial cells in the CNS. In addition, it has been recently reported that ZIKV infection of microglia cells leads to modulation of the expression of numerous metabolites, such as phosphatidylcholine, phosphatidylserine, ceramide and sphingomyelin, and carboxylic acids. Some of these metabolites are involved in neuronal differentiation, regulation of apoptosis, virion architecture, and viral replication. In the same study, ZIKV infection was associated with concomitant secretion of inflammatory mediators linked to CNS inflammation and alterations in endothelial and microvascular permeability, such as IL-6, TNF-α, IL-1β, iNOS, and NO. Furthermore, ZIKV infection resulted in upregulation of the expression of the gene encoding for CX3CR1, a chemokine receptor known to regulate functional synapse plasticity and signaling between microglial cells (Diop et al., 2018). Upregulation of these proinflammatory mediators is important because inflammatory Th1 cytokines, such as TNF-α, IL-1β, IL-6 and CCL-2 play important roles in increasing endothelial/microvascular permeability, both *in vivo* and *in vitro* (Toborek et al., 1995; Nguyen et al., 2003; Fahey and Doyle, 2019). IFN-γ, on the other hand, tends to stabilize TJs (Capaldo and Nusrat, 2009). Consistent with these results, our analyses performed on microvessels isolated from ZIKV-infected brains also revealed upregulation of genes involved in immune responses against viral infection (Figure 7). In addition, we observed downregulation of selected genes related to neurogenesis and cell differentiation, namely, Omp and Plekhb1. Intriguing further studies are needed to determine if these gene alterations might influence the development of neuronal division and growth, leading to microcephaly in offspring. While the gene expression changes observed in the present study were independent of the infection by individual ZIKV strains, detailed genetic studies of different strains may reveal viral genetic determinants affecting ZIKV-mediated input on TJ and BBB functionality as well as on CNS neuroinvasion.

By studying different strains of ZIKV, we aimed to determine possible differences in viral tropism that lead to brain infection and neuropathogenesis. We found no significant difference between African and Asian strains regarding viral replication kinetics, viral burden, and neurotropism, with approximately 40–50% of infected animals having detectable ZIKV RNA in their brain tissue (Figure 4). These results highlight the importance of studies on immunocompetent animals, as experiments performed on immunocompromised animals obtained conflicting results, associating African strains with worse disease progression (Dowall et al., 2017; Simonin et al., 2017; Tripathi et al., 2017).

In conclusion, our results indicate that ZIKV penetrates the brain parenchyma early after infection resulting in brain infection of approximately 40–50% of infected immunocompetent mice. However, increased permeability of the BBB does not appear to be the major mechanism for CNS entry of ZIKV, though changes in TJ protein expression and strain-dependent disturbances in BBB permeability were observed.

## Data Availability

All datasets generated for this study are included in the manuscript/supplementary files.

## Ethics Statement

All animal procedures were approved by the University of Miami Institutional Care and Use Committee (IACUC) in accordance with the National Institutes of Health (NIH) guidelines.

## Author Contributions

AL designed and performed all the experiments, analyzed the data, and wrote the manuscript. IA and LB contributed to generation of the data. NE-H and MN conceived and designed the studies. MT conceived and designed the studies, and wrote the manuscript.

## Conflict of Interest Statement

The authors declare that the research was conducted in the absence of any commercial or financial relationships that could be construed as a potential conflict of interest.
